# Enhancement of the anthocyanin contents of *Caladium* leaves and petioles via metabolic engineering with co-overexpression of AtPAP1 and ZmLc transcription factors

**DOI:** 10.3389/fpls.2023.1186816

**Published:** 2023-06-21

**Authors:** Ximeng Yang, Airong Li, Jing Xia, Yan Huang, Xin Lu, Gangyu Guo, Shunzhao Sui

**Affiliations:** Chongqing Engineering Research Center for Floriculture, Key Laboratory of Horticulture Science for Southern Mountainous Regions of Ministry of Education, College of Horticulture and Landscape Architecture, Southwest University, Chongqing, China

**Keywords:** *Caladium bicolor*, *AtPAP1*, *ZmLc*, anthocyanin biosynthesis, metabolic engineering

## Abstract

**Introduction:**

Metabolic engineering of anthocyanin synthesis is an active research area for pigment breeding and remains a research hotspot involving AtPAP1 and ZmLc transcription factors. *Caladium bicolor* is a desirable anthocyanin metabolic engineering receptor, with its abundant leaf color and stable genetic transformation system.

**Methods:**

We transformed *C. bicolor* with *AtPAP1* and *ZmLc* and successfully obtained transgenic plants. We then used a combination of metabolome, transcriptome, WGCNA and PPI co-expression analyses to identify differentially expressed anthocyanin components and transcripts between wild-type and transgenic lines.

**Results:**

Cyanidin-3-*O*-glucoside, cyanidin-3-*O*-rutinoside and peonidin-3-*O*-rutinoside are the main components of anthocyanins in the leaves and petioles of *C. bicolor*. Exogenous introduction of *AtPAP1* and *ZmLc* resulted in significant changes in pelargonidins, particularly pelargonidin-3-*O*-glucoside and pelargonidin-3-*O*-rutinoside in *C. bicolor*. Furthermore, 5 MYB-TFs, 9 structural genes, and 5 transporters were found to be closely associated with anthocyanin synthesis and transport in *C. bicolor*.

**Discussion:**

In this study, a network regulatory model of AtPAP1 and ZmLc in the regulation of anthocyanin biosynthesis and transport in *C. bicolor* was proposed, which provides insights into the color formation mechanisms of *C. bicolor*, and lays a foundation for the precise regulation of anthocyanin metabolism and biosynthesis for economic plant pigment breeding.

## 1 Introduction

Anthocyanins are important water-soluble pigments that impart various colors to vegetative and reproductive plant organs (Yan et al., 2021). These colors, primarily red, purple, and blue, are regulated by anthocyanins through different biosynthetic pathways, which pigment flowers, fruits, and seeds to attract pollinators and seed dispersers (Chen et al., 2019; Mattioli et al., 2020). In addition, anthocyanins help protect plants against cold or drought, and defend against herbivores and pathogens (Ma and Constabel, 2019). As a result, anthocyanin-rich plants are often used in landscape gardens to enhance the natural landscape (Jiang et al., 2016; Sun et al., 2020). Therefore, anthocyanins have multiple functions for plant survival and are highly commercially valuable.

Anthocyanins belong to the diverse family of aromatic molecules known as flavonoids (Belwal et al., 2020). Plants contain structural genes from the anthocyanin biosynthesis pathway, including phenylalanine ammonialyase (PAL), cinnamate 4-hydroxylase (C4H), 4-coumarate-CoA ligase (4CL), chalcone synthase (CHS), chalcone isomerase (CHI), flavanone 3-hydroxylase (F3H), flavonoid 3’ -hydroxylase (F3’ H) or flavonoid 3’,5’-hydroxylase (F3’5’H), dihydroflavonol 4-reductase (DFR), anthocyanidin synthase (ANS), and anthocyanidin-*O* -methyltransferase (AOMT) (Saigo et al., 2020). To be stored, anthocyanin must be efficiently transported from the surface of the endoplasmic reticulum to the vacuole. Four transporters related to anthocyanin transport have been identified, including glutathione S-transferase (GST), multidrug resistance-associated protein (MRP, an ABCC transporter-type), multidrug and toxic compound efflux (MATE) transporters, and the plasma membrane H^+^-ATPase (AHA10) (Shi and Xie, 2014). In addition, the MYB-bHLH-WD40 complex (MBW), consisting of R2R3-MYB, bHLH, and WD40 proteins, directly regulates the transcription of the anthocyanidins structural genes and transporter genes (Lloyd et al., 2017; Xu et al., 2020). That is, transcriptionally activating MYB transcription factors usually lead to anthocyanin accumulation in plant leaves or fruits (Naing and Kim, 2018; Xie et al., 2020). MYB transcription factors regulating anthocyanin biosynthesis in the model plant *Arabidopsis thaliana* have been studied most clearly, among which AtPAP1 (Production of Anthocyanin Segment 1) is the key transcription factor regulating anthocyanin biosynthesis. Activating excessive expression of *AtPAP1* induces a large accumulation of anthocyanins, leading to a deep purple color in *A. thaliana* leaves (Borevitz et al., 2000). Overexpression of *AtPAP1* affects the expression of genes such as *CHS*, *CHI*, and *ANS* (Tohge et al., 2005). The *Lc* (Leaf color) gene encodes a bHLH-type transcription factor from maize, one of the earliest discovered bHLH family transcription factors which are important for regulating anthocyanin production (Ludwig et al., 1989). *ZmLc* can complement the anthocyanin deficient phenotype in the double mutant of *Petunia hybrida an2/an11* by activating the expression of *CHS* and *DFR* (Quattrocchio et al., 1993).

Metabolic engineering of anthocyanin synthesis remains a research hotspot involving AtPAP1 and ZmLc transcription factors. Heterologous expression of *AtPAP1* in *Nicotiana benthamiana* (tobacco) can promote the accumulation of anthocyanins (Gatica-Arias et al., 2012; Qiu et al., 2014). Transferred the *AtPAP1* into tobacco and detected a medicinal anthocyanin Cyanidin-3-*O*-rutinoside in the transgenic tobacco, which accounts for 98% of the total anthocyanin content (He et al., 2017). Moreover, overexpression *AtPAP1* can effectively induce anthocyanin production in *Rosa hybridaby*, *Salvia miltiorrhiza, Solidago Canadensis, Solanum nigrum* and *Scutellaria baicalensis* (Zvi et al., 2012; Zhang et al., 2014b; Skaliter et al., 2019; Chhon et al., 2020; Ha Park et al., 2021). In addition, ectopic expression of *ZmLc* has been shown to enhance anthocyanin accumulation in *Solanum lycopersicum* (Goldsbrough et al., 1996), *P. hybrida* (Bradley et al., 1998), *Medicago sativa* (Ray et al., 2003), *C. bicolor* (Li et al., 2005), *Malus domestica* (Li et al., 2007), and *S. baicalensis* (Ha Park et al., 2021). In *A. thaliana*, expressing *AtPAP1* and *ZmLc* simultaneously increased anthocyanin production (Sharma and Dixon, 2005). Co-expression of *AtPAP1* and *ZmLc* was transferred into the rare wild medicinal plant *Saussurea involucrata*, and at least 4 derivatives of cyanidins were found in the callus and tender shoots of the transgenic strain; At the same time, the expression of most structural genes in the anthocyanin synthesis pathway is induced, with the most significant change in the expression level of the *CHS* (Qiu et al., 2013).

*Caladium bicolor* (Araceae family) is a popular choice for containers or as accent/border plants due to its attractive and variably shaped leaves (Li et al., 2005). Color is an important trait of ornamental plants that has a direct influence on their ornamental and economic value (Boutigny et al., 2020). *C. bicolor* has a diverse range of colors, and the formation of color patches is closely linked to changes in anthocyanin synthesis and content. *C. bicolor* has many varieties that provide a great resource for identifying anthocyanins and anthocyanin-related genes. Furthermore, *C. bicolor* has a reliable genetic transformation system, making it an ideal model plant for studying anthocyanin metabolism. This transgenic host also supports targeted breeding through genetic engineering, providing a basis for the cultivation of new varieties with desired colors for specific garden applications.

This study successfully transferred *AtPAP1* and *ZmLc* into *C. bicolor* and plants, wild-type (WT) and transgenic-type (TT), were subjected to metabolome, transcriptome, WGCNA and PPI co-expression network analysis. The aim of this study was to quantify the composition and content of anthocyanins in *C. bicolor*, and to investigate the regulation of *AtPAP1* and *ZmLc* expression in *C. bicolor*, providing a basis for metabolic engineering of anthocyanin biosynthesis.

## 2 Methods

### 2.1 Plant materials

**Figure 1:**
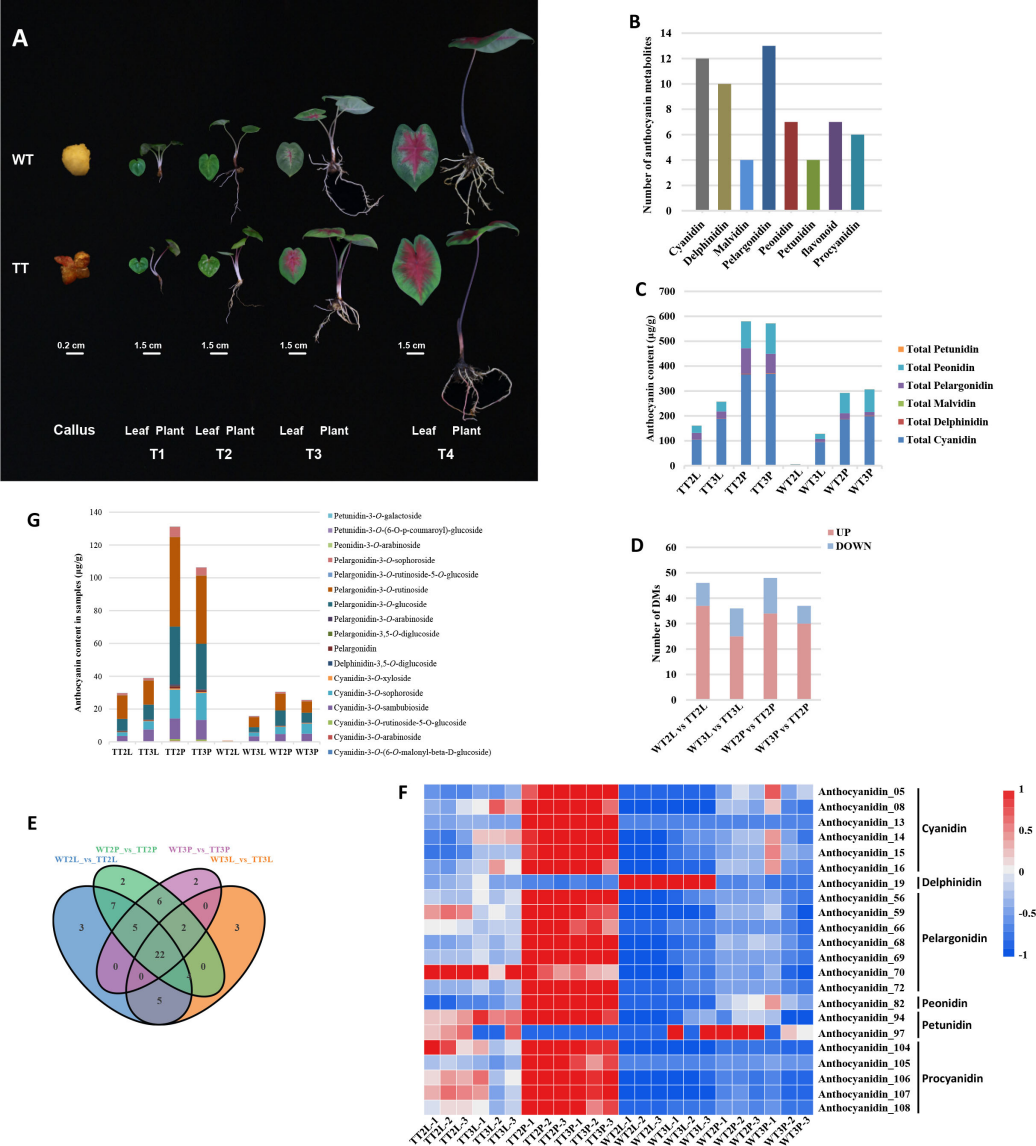
Phenotype and differentially accumulated metabolites analysis of metabolome in TTs and WTs. **(A)**, The phenotype of callus, roots, petioles, leaves of transgenic lines and WT. T1-T4, representing four growth periods of *C. bicolor*. **(B)**, Classification and statistical analysis of all metabolites detected. **(C)**, The relative content of anthocyanins. **(D)**, Number of DMs among all comparison units. **(E)**, Venn analysis among WT2L vs. TT2L, WT2P vs. TT2P, WT3L vs. TT3L and WT3P vs. TT3P. **(F)**, The heatmap analysis of overlapping DMs according to the relative content in samples. **(G)**, The relative content of overlapping different anthocyanins.

*C. bicolor* seedlings were collected from a nursery at Southwest University, Chongqing, China. In stage 2 (T2), white spots appeared on the leaves of the wild-type plants after plantlets were transferred to soil, whereas red spots appeared on the transgenic-type plants. In stage 3, red spots were seen on the wild type, and the transgenic type’s red spot area covered half the surface area of the leaf. The transgenic-AtPAP1+ZmLc-type and wild-type plants’ leaves (L) and petioles (P), respectively, were sampled at stages T2 and T3 of the *C. bicolor* plant. The material was then marked with the notations WT2L, WT2P, WT3L, WT3P, TT2L, TT2P, and TT3L for metabolome and transcriptome analysis with three biological replicates (**Figure 1A**).

### 2.2 Plant transformation

The State Key Laboratory for Conservation and Utilization of Subtropical Agro-Bioresources, Guangzhou, China, provided the plant binary expression vector (pC1300-*PAP1*+*Lc*) (**Supplementary Method 1**). The vector constructs were introduced into *Agrobacterium tumefaciens* strain GV3101 and used to transform *C. bicolor* using the leaf disk transformation–regeneration method (Li et al., 2005). *C. bicolor* tender leaves as the explant were inoculated into callus induction Murashige and Skoog Medium (MS) with 4 mg L^−1^ 6-benzylaminopurine (6-BA), 0.2 mg L^−1^ α-Naphthaleneacetic acid (NAA) for 2 days, and the explants were immersed in pC1300-*PAP1+Lc Agrobacterium* solution (OD600 = 0.3) with 2 mg L^−1^ acetosyringone (AS) for 5 minutes. The infected explants were inoculated into callus induction medium for 2 days in dark culture at 28°C. Inoculated into MS with 4 mg/L 6-BA, 0.2 mg L^−1^ NAA, 7.5 mg L^−1^ hygromycin (Hyg) and 500 mg L^−1^ carbenicillin (Carb) for selection. The explants were cultured in a growth chamber at 25 ± 1°C with 16-h photoperiod under cool white light (60 µmol m^-1^ s^−1^) and subcultured on fresh medium every 20 days. After the infected leaf-disc grew callus, were transferred to MS medium supplemented with 2 mg L^−1^ 6-BA and 0.2 mg L^−1^ NAA. Shoots of about 1.5 cm in length, were transferred to a hormone-free medium for rooting (1/2 MS medium supplemented with 600 mg L^−1^ NAA). Rooted plantlets 3-4 cm high were transferred to soil for further growth in a greenhouse.

### 2.3 Identification and quantitative analysis of metabolite

The sample was freeze-dried, ground into powder (30 Hz, 90 s). 50 mg powder was weighted and extracted with 0.5 mL methanol/water/hydrochloric acid (500:500:1, V/V/V). Subsequently, the extract was vortexed for 5 min and ultrasound for 5 min and centrifuged at 12, 000 g under 4°C for 3 min. After repeating the extractions twice, the supernatants were collected and filtrated through a membrane filter (SCAA-104, 0.22 μm pore size; ANPEL, Shanghai, China, http://www.anpel.com.cn/) before analysis.

The sample extracts were analyzed using an UPLC-ESI-MS/MS system (UPLC, ExionLC™ AD, https://sciex.com.cn/; MS, Applied Biosystems 6500 Triple Quadrupole, https://sciex.com.cn/). The analytical conditions were as follows, UPLC: column, WatersACQUITY BEH C18 (1.7 µm, 2.1 mm*100 mm); the mobile phase consisted of solvent A, pure water with 0.1% formic acid, and solvent B, methanol with 0.1% formic acid. Sample measurements were performed with a gradient program that employed the starting conditions of 95% A and 5% B. A linear gradient of 50% A and 50% B was programed at 6 min. Within 12 min, a linear gradient of 5% A and 95% B was programed, and a composition of 5% A and 95% B was maintained for 2 min. Subsequently, a composition of 95% A and 5.0% B was adjusted within 14 min and kept for 2 min. The flow rate was set to 0.35 mL per minute; The column oven temperature was set to 40 °C, and the injection volume was 2 μL. The effluent was alternatively connected to an ESI-triple quadrupole linear ion trap (QTRAP)-MS system.

Linear ion trap (LIT) and triple quadrupole (QQQ) scans were acquired on a triple quadrupole-linear ion trap mass spectrometer (QTRAP), QTRAP^®^ 6500+ LC-MS/MS System, equipped with an ESI Turbo Ion-Spray interface, operating in positive ion mode and controlled by Analyst 1.6.3 software (Sciex). The ESI source operation parameters were as follows: ion source, ESI+; source temperature 550 °C; ion spray voltage (IS) 5500 V; curtain gas (CUR) was set at 35 psi, respectively.

Anthocyanins were analyzed using scheduled multiple reaction monitoring (MRM). Data acquisitions were performed using Analyst 1.6.3 software (Metware Biotechnology Co., Ltd., Wuhan, China). The differentially accumulated anthocyanins were identified by R statistical software (www.r-project.org), and the screening criteria were as follows: significantly regulated metabolites between groups were determined by absolute |Log_2_FC (fold change)| ≥1.

### 2.4 Illumina sequencing and data analysis

Total RNA was extracted from three replications of TT and WT plants leaves and petioles at T2 and T3 using the Quick RNA isolation Kit (Huayueyang, Beijing, China) following the manufacturer’s protocols. The *C. bicolor* RNA-seq transcriptome library was prepared following Illumina^®^ Stranded mRNA Prep, Ligation from Illumina (San Diego, CA) using 1μg of total RNA. The raw paired end reads were trimmed and quality controlled by fastp with default parameters (Chen et al., 2018). Then clean data from the samples of *C. bicolor* were used to do *de novo* assembly with Trinity (Grabherr et al., 2011). BLAST2GO program was used to get GO annotations of unique assembled transcripts for describing biological processes, molecular functions and cellular components (Conesa et al., 2005). Metabolic pathway analysis was performed using the Kyoto Encyclopedia of Genes and Genomes (KEGG) (Kanehisa and Goto, 2000).

To identify differential expression genes (DEGs) between two different samples (WT2L vs. TT2L, WT2P vs. TT2P, WT3L vs. TT3L and WT3P vs. TT3P), the expression level of each gene was calculated according to the transcripts per million reads (TPM) method. RSEM (Li and Dewey, 2011) was used to quantify gene abundances. Essentially, differential expression analysis was performed using the DESeq2 (Love et al., 2014), DEGs with |log_2_ (fold change)| ≥ 1 and P-adjust ≤ 0.05 were considered to be significantly different expressed genes. In addition, functional-enrichment analysis including GO and KEGG were performed to identify which DEGs were significantly enriched in GO terms and metabolic pathways at P-adjust ≤ 0.05 compared with the whole-transcriptome background. GO functional enrichment and KEGG pathway analysis were carried out by Goatools and KOBAS (Xie et al., 2011).

### 2.5 Co-expression network analysis

The R statistical software, DCGL, was used to identify the key candidate gene clusters using Weighted Gene Co-expression Network Analysis (WGCNA) (Langfelder and Horvath, 2008). The modules were obtained using the automatic network construction function. Modules were identified using default settings, except that the soft power was 8, the min module size was 30, and the merge cut height was 0.25. The genes of the connections between nodes with weight values greater than 0.4 were chosen to construct a visualizing co-expression network. Pearson correlation coefficients and P-values were calculated for the transcriptome TPM and metabolomics (the content of anthocyanins) data integration using Spearman’s method. Correlations corresponding to a coefficient of |R| >0.7 were selected to construct a visualizing co-expression network. Next, the string database (http://string-db.org/) was used to perform protein-protein interaction network analysis on the genes of interest (Doncheva et al., 2018). For *A. thaliana* having a relatively comprehensive protein interaction relationship in the database, the interaction relationship corresponding to the genes of interest in our research was directly extracted from the database to construct the network. Cytoscape software 2.8 was used to visualize the co-expression networks (Doncheva et al., 2018).

### 2.6 Isolation and sequence analyses of *MYBs*

The sequences of *MYB-5* and *MYB-10* genes were obtained from transcriptome and identified by Co-expression Network Analysis, which were translated using the NCBI Open Reading Frame Finder and sequenced using DNAMAN. R2R3-MYBs amino acid sequences from other plants were obtained from GenBank. All protein sequences are listed in **Table S11**. The protein sequence alignment was generated by ClustalW algorithm in MEGA 7 (Kumar et al., 2016) and used for phylogenetic analyses. A maximum likelihood (ML) tree was constructed, and branch supports were estimated using 1000 bootstrap replicates. Bootstrap values greater than 50 are shown along the branches.

### 2.7 Reverse transcription quantitative PCR (qRT-PCR) analysis

An aliquot of cDNA synthesized from the total RNA samples for Illumina sequencing was reverse transcribed using the NOVA All-in-one First-Strand Synthesis Master kit (Yugong Biolabs, Jiangsu, China). Primer 5.0 (**Table S1**) designed the primers for the selected genes with the *UBC* gene (Pan, 2021) as the reference for normalization. Quantitative real-time PCR was performed using the 2×TSINGKE^®^ Master qPCR Mix (SYBR Green I with UDG) (Tsingke, Beijing, China) on a CFX96 Real-time PCR System (Bio-Rad, CA, USA). The program involved initial denaturation at 50 °C for 2 min, then 95 °C for 2 min, followed by 40 cycles of 95 °C for 15 s, and 60 °C for 30 s. Each plate was amplified three times, and the 2^-ΔΔCT^ method was used to analyze gene expression levels (Schmittgen and Livak, 2008).

## 3 Results

### 3.1 Metabolic differences among the transgenic lines (TTs) and wild-type lines (WTs)

Co-overexpression of *AtPAP1* and *ZmLc* caused a different color formation in various plant parts (**Figure 1A**). The metabolites were detected by UHPLC-ESI-MS/MS to further analyze the differences in metabolites in the *C. bicolor* leaves and petioles at T2 and T3 of different colors. A total of 63 metabolites were identified from these eight sample groups. The annotation, relative content, and classification of the 63 metabolites are detailed in **Table S2**. These metabolites were classified into 8 categories, including cyanidin, delphinidin, malvidin, pelargonidin, peonidin, petunidin, flavonoid and procyanidin. And these categories contained 4 to 13 types of metabolites (**Figure 1B**). The content of cyanidin, delphinidin, malvidin, pelargonidin, peonidin, and petunidin metabolites were calculated separately. Among these six major anthocyanidins, the contents of cyanidin, peonidin, pelargonidin, delphinidin, malvidin, petunidin were in descending order (**Figure 1C**).

Based on the data obtained from the metabolome, 46, 36, 48 and 37 types of Different Metabolites (DMs) were identified between the WT2L vs. TT2L, WT2P vs. TT2P, WT3L vs. TT3L and WT3P vs. TT3P groups, respectively (**Figure 1D**). A total of 61 DMs were identified among all samples **Table S3**. Moreover, the overlapping metabolites were identified. The results showed that 22 DMs were shared among WT2L vs. TT2L, WT2P vs. TT2P, WT3L vs. TT3L and WT3P vs. TT3P (**Figure 1E**). These 22 metabolites included 17 of anthocyanins and 5 proanthocyanidins and the relative contents of these 22 DMs were illustrated in a heatmap (**Figure 1F**). The classification results of these 17 anthocyanins indicated that most were pelargonidin-3-*O*-glucoside, pelargonidin-3-*O*-rutinoside, and then cyanidin-3-*O* -sambubioside, cyanidin-3-*O* -sophorosid, and a small amount were delphinidin, malvidin and petunidin (**Figure 1G**).

### 3.2 *De novo* sequencing, assembly, and identification of differentially expressed genes

To further analyze the molecular mechanism of TT and WT plants, transcriptome sequencing was used to analyze the leaves (L) and petiole (P) of transgenic material and wild-type material at the T2 and T3 stages. These samples were used to construct 24 sequencing libraries (three biological replicates). A total of 177.98 Gb clean data was obtained. Each sample had more than 6.21 Gb of clean data. The percentage of Q30 base was more than 93.71% (**Table S4**). The clean data were assembled, then the assembly results were optimized and evaluated. A total of 129980 unigenes and 268446 transcription factors (TFs) were obtained *via* assembly (average length of N50: 1357bp) (**Table S5**). The clean reads were compared with the assembled reference sequence using Trinity to obtain the mapping results of each sample. The analysis alignment rate ranged from 60.20% to 67.27% (**Table S6**).

**Figure 2:**
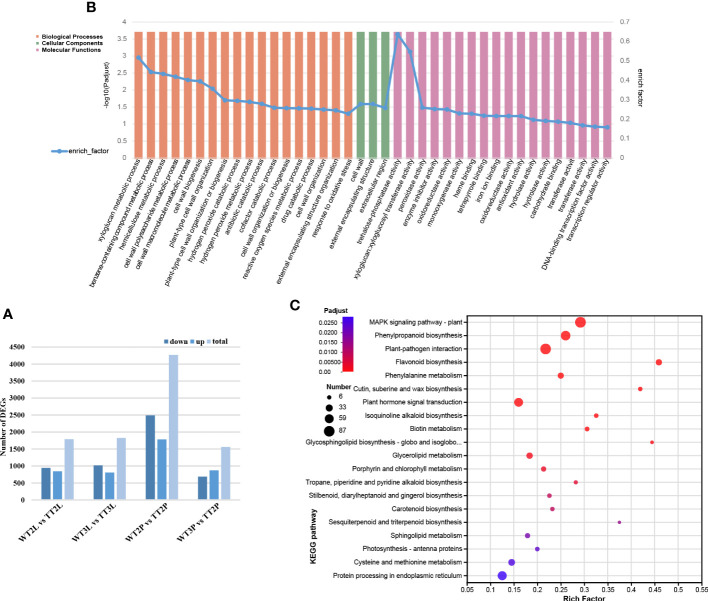
Differentially expressed unigenes (DEGs) in TTs and WTs. **(A)**, The number of up- and down-regulated genes in WT2L vs. TT2L, WT2S vs. TT2S, WT3L vs. TT3L, WT3S vs. TT3S, and WT2L vs. WT3L. **(B)**, GO enrichment analysis. **(C)**, KEGG enrichment analysis.

Transcriptomic analyses of WT2L vs. TT2L, WT2P vs. TT2P, WT3L vs. TT3L and WT3P vs. TT3P were conducted to identify the key DEGs related to the color formation (**Figure 2A**). A total of 7070 DEGs were identified based on a 2-fold change at P < 0.05 (**Table S7**).

The function of the DEGs was determined using Gene Ontology (GO) enrichment analysis. A total of 7070 DEGs were related to biological processes (BP), cellular components (CC), and molecular functions (MFs). There were 39 most enriched GO terms, of which 18 belonged to BP, 3 CC as well as 18 MF. The most enriched GO terms in these three categories were listed in **Figure 2B**. KEGG enrichment analysis was used for pathway classification and functional enrichment to further evaluate the biological functions of the identified DEGs. Significant enrichment of DEGs in phenylpropanoid biosynthesis (ko00940) and flavonoid biosynthesis (ko00941) pathways (**Figure 2C**).

### 3.3 WGCNA and functional annotation of DEGs-related anthocyanin synthesis

**Figure 3:**
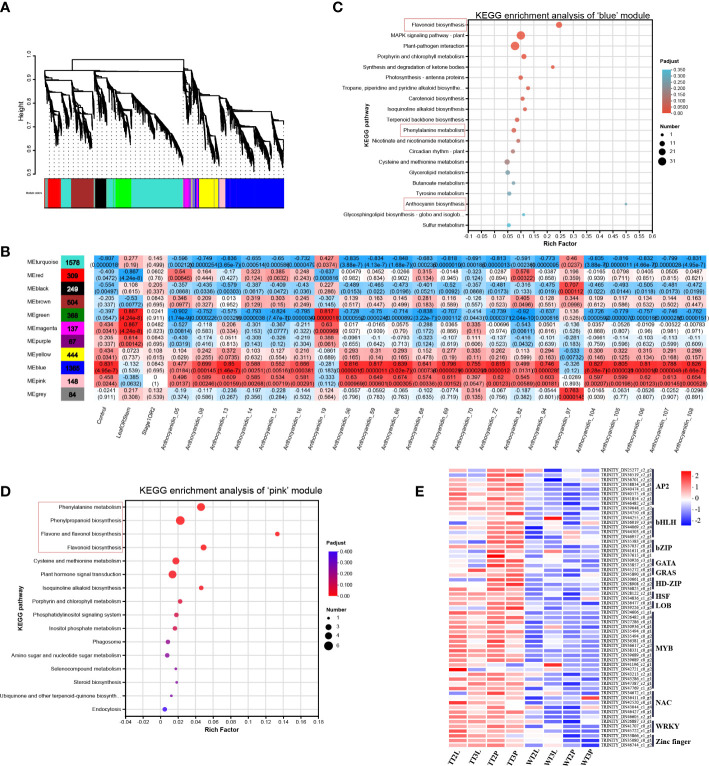
Weighted gene co-expression network analysis. **(A)**, Hierarchical clustering tree (cluster dendrogram) results showed 11 expression modules, labeled with different colors. **(B)**, Module correlations and corresponding p-values. The left panel shows the 11 modules and the number of genes in each module. The value inside each box represents Pearson’ s correlation coefficient between the module with anthocyanin, and the number in each parentheses represents p-value. The color scale on the right represents the degree of correlation between modules and anthocyanins and the red represent high correlation. **(C)**, KEGG enrichment analysis of genes in ‘blue’ module. **(D)**, KEGG enrichment analysis of genes in ‘pink’ module. **(E)**, The heatmap of identified TFs in ‘blue’ and ‘pink’ modules.

WGCNA is used to describe the correlations patterns of gene expression and reveal clusters or modules of genes whose expressions are highly correlated. The module eigen genes, intramodular hub genes and the association between modules with one another and sample traits can be determined using WGCNA (Langfelder and Horvath, 2008). Herein, WGCNA was conducted using the TPM values of the total of 7070 DEGs and the content of the overlapping 22 DMs as source data (**Figure 3A**), identified 11 co-expression modules, where the ‘blue’ and ‘pink’ modules were significantly correlated with the transgenic line and anthocyanin content (**Figure 3B**). Furthermore, the biological functions of the ‘blue’ (1365 DEGs) and ‘pink’ (148 DEGs) modules were analyzed using KEGG pathway enrichment analysis (**Figures 3C, D** **)**. The ‘blue’ and ‘pink’ modules were both enriched in the ‘flavonoid biosynthesis’ and ‘phenylalanine metabolism’ pathway. The genes related to ‘anthocyanin biosynthesis’ and ‘phenylpropanoid biosynthesis’, ‘flavone and flavonol biosynthesis’ were also clustered in ‘blue’ and ‘pink’ modules, respectively (**Figures 3C, D** **)**.

The ‘blue’ and ‘pink’ modules contained 59 TFs, 32 structural genes and 24 transporters that may be related to anthocyanin synthesis and transport. The 115 DEGs in the ‘blue’ and ‘pink’ modules were then analyzed (**Table S8**).

**Figure 4:**
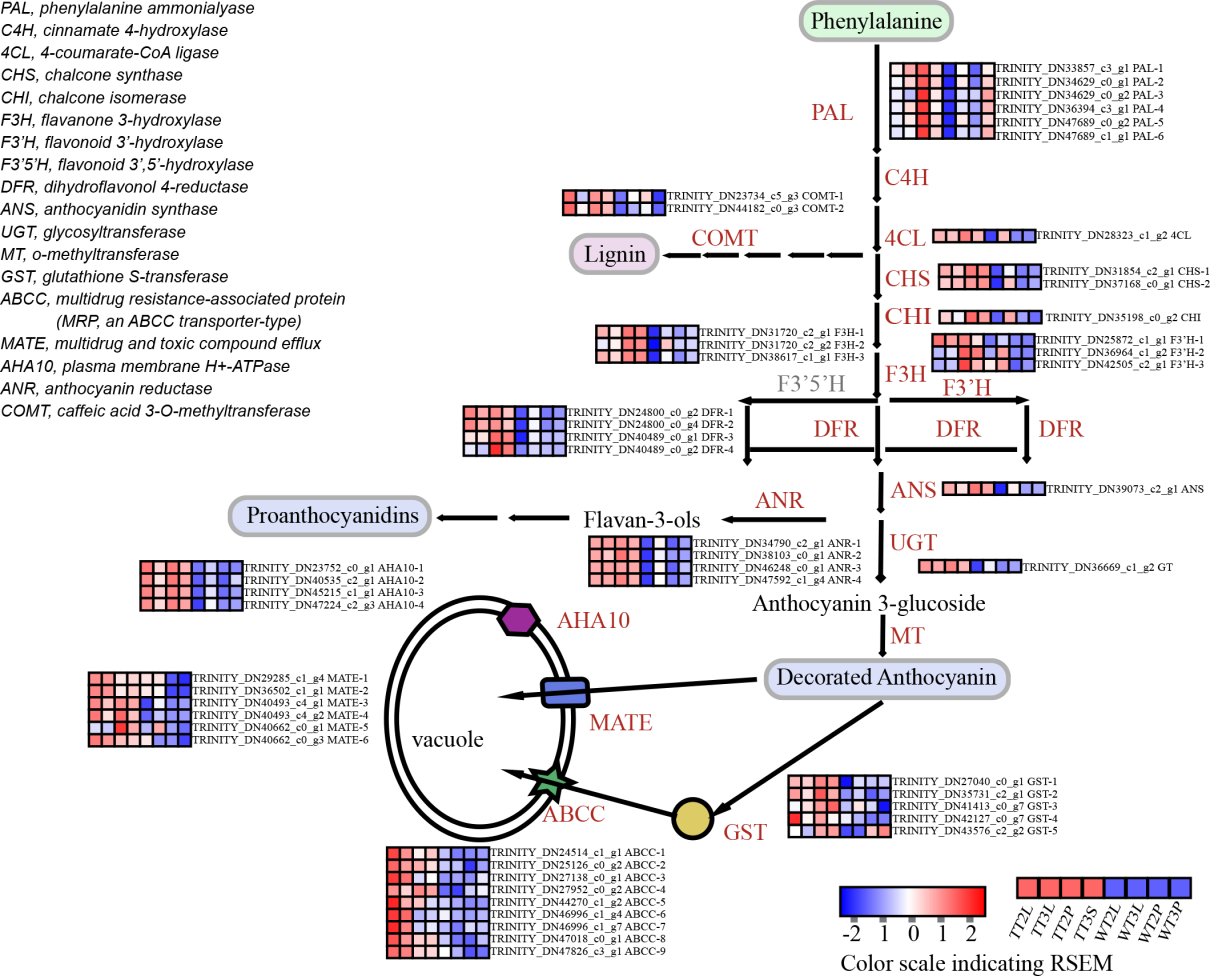
The identification of DEGs in ‘blue’ and ‘pink’ modules of anthocyanin biosynthesis and transport pathway.

In ‘blue’ and ‘pink’ modules identified 32 DEGs in the anthocyanin synthesis pathway (**Figure 4**; **Table S8**), including the phenylpropanoid biosynthesis, flavonoid biosynthesis, and anthocyanin biosynthesis pathways. The 24 transporters were identified by the annotation results of the DEGs in the ‘blue’ and ‘pink’ modules, which belonged to the MATE, AHA10, ABCC and GST, that may be related to anthocyanin transport (**Figure 4**; **Table S8**). The ‘blue’ and ‘pink’ modules contained 59 TFs, the classified results indicated that these TFs belonged to the AP2/ERF, bHLH, bZIP, GATA, GRAS, HD-ZIP, HSF, LOB, MYB, NAC, WRKY and Zinc finger family (**Figure 3E**; **Table S8**).

### 3.4 Network analysis of the transcriptome and metabolomics

**Figure 5:**
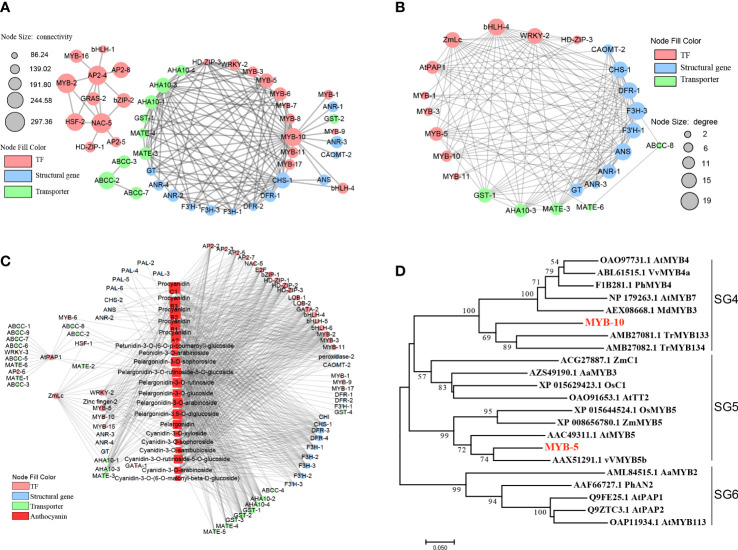
Connection network between KEY DEGs and differential metabolites. **(A)**, Gene co-expression network of key genes by WGCNA analysis. **(B)**, Co-expression network of key genes and anthocyanins content by Pearson analysis. **(C)**, Gene co-expression network of key genes by PPI analysis. The circle sizes represent the number of connections between genes. The red, blue and green circles represent transcription factors, structural genes of anthocyanin biosynthesis and transporters, respectively. **(D)**, Phylogenetic relationships of MYB-TFs. The MYBs belonging to the SG4-SG6 and R3-MYB were used to build a phylogenetic tree based on maximum likelihood, which was employed to infer the MYB candidates.

Further narrow the scope of key genes, 59 TFs, 32 structural genes and 24 transporters in the ‘blue’ and ‘pink’ modules were visualized using Cytoscape version 2.8, filtering the connections between nodes with weight values greater than 0.4 for analysis (**Figure 5A**; **Table S9**). We extracted 33 genes. And MYB-10, CHS-1 and AHA10-3 were identified as hub genes, which not only were putatively associated with transgenic line and anthocyanin contents and closely related to genes in the anthocyanin synthesis pathway (**Figure 5A**). Phylogenetic analysis indicated that MYB-10 belonged to the subgroup 4 of the R2R3-MYB transcription factor gene family (**Figure 5D**). Although the weight values of MYB-2, AP2-4 and NAC-5 were also high, they formed a cluster of their own and were not clustered with the genes related to anthocyanin biosynthesis (**Figure 5A**).

Moreover, the interaction network diagram was constructed using the TPM between the key DEGs (59 TFs, 32 structural genes, 24 transporters, *AtPAP1* and *ZmLc*) and the content of the overlapping 17 anthocyanins and 5 proanthocyanidins, for directed displaying the AtPAP1 and ZmLc regulatory network of anthocyanin accumulation. Correlations corresponding to a coefficient of |R| >0.7 were selected to visualize co-expression network (**Figure 5C**). There were 26 genes directly associated with AtPAP1 and ZmLc. Analysis of the DMs and genes directly related to AtPAP1 and ZmLc revealed that both were significantly correlated with pelargonidin-3-*O*-rutinoside-5-*O*-glucoside. In addition, ZmLc was directly associated with petunidin-3-*O*-(6- *O* -p-coumaroyl)-glucoside, procyanidin A2, procyanidin B2 and procyanidin B3, while AtPAP1 was indirectly related to procyanidin A2 through MYB-6 and HSF-1. Furthermore, ZmLc was also found to be connected to other type of cyanidin, pelargonidin and peonidin through other genes, with MYB-5 and WRKY-2 being important intermediate bridges (**Figure 5B**). Phylogenetic analysis revealed that MYB-5 was in the branch which belonged to the subgroup 5 of the R2R3-MYBs (**Figure 5D**).

Herein, the 33 genes extracted by WGCNA analysis method were compared with 26 genes obtained by Pearson correlation analysis, as well as *AtPAP1* and *ZmLc* genes, a total of 51 genes. Subsequently, these 51 genes were analyzed using PPI to further evaluate the regulatory network of anthocyanin synthesis and transport pathway in *C. bicolor*, and how AtPAP1 and ZmLc regulate anthocyanin synthesis and transport pathway in *C. bicolor* (**Figure 5C**). AtPAP1 and ZmLc were found to have expression patterns closely related to key structural genes and transporters, such as CHS-1, DFR-1, F3H-3, F3’H-1, ANS, ANR-1, GT, GST-1, AHA10-3 and MATE-3. Not only that, they are both closely related to transcription factors WRKY-2. AtPAP1 is correlated with bHLH-4, while ZmLc is also associated with Zinc finger, HD-ZIP and MYB-like transcription factors. Except for AtPAP1, the expression patterns of 5 MYB TFs, especially MYB-5 and MYB-10 were closely related to those of key structural genes and transporters (**Figure 5C**). The final screen yielded 5 MYB-TFs, 9 structural genes, and 5 transporter key genes (**Table 1**; **Table S10**).

**Table 1:** The candidate genes involved in AtPAP1 and ZmLc regulating anthocyanin synthesis and transport in *C. bicolor*.

Gene ID	Annotion	NR_hit-name	length	KO_id
TRINITY_DN24606_c1_g1	MYB-1	XP_012479075.1	268	K09422
TRINITY_DN27286_c0_g1	MYB-3	XP_018677400.1	1148	K09422
TRINITY_DN35494_c0_g1	MYB-5	XP_008791485.1	1329	K09422
TRINITY_DN39689_c0_g1	MYB-10	XP_021623909.1	1416	K09422
TRINITY_DN39689_c0_g2	MYB-11	XP_014508008.1	988	K09422
TRINITY_DN44182_c0_g3	CAOMT-2	XP_023885967.1	355	K13066
TRINITY_DN31854_c2_g1	CHS-1	ABE01413.1	1195	K00660
TRINITY_DN24800_c0_g2	DFR-1	AAP20866.1	1647	K13082
TRINITY_DN38617_c1_g1	F3H-3	AAP20865.1	1684	K00475
TRINITY_DN25872_c1_g1	F3’H-1	AHL83556.1	2975	K05280
TRINITY_DN39073_c2_g1	ANS	AAP20867.1	1757	K05277
TRINITY_DN34790_c2_g1	ANR-1	XP_010267828.1	798	K21102; K08695
TRINITY_DN46248_c0_g1	ANR-3	EES11483.1	306	K08695
TRINITY_DN36669_c1_g2	GT	XP_010913632.1	1834	K12930
TRINITY_DN47018_c0_g1	ABCC-8	XP_010915725.1	4529	K05666
TRINITY_DN45215_c1_g1	AHA10-3	XP_024021501.1	3775	K01535
TRINITY_DN27040_c0_g1	GST-1	KMZ75853.1	1009	K00799
TRINITY_DN40493_c4_g1	MATE-3	XP_018731884.1	1291	K03327
TRINITY_DN40662_c0_g3	MATE-6	XP_010930069.1	1508	K03327

### 3.5 qRT -PCR analysis of DEGs involved in anthocyanin biosynthesis

**Figure 6:**
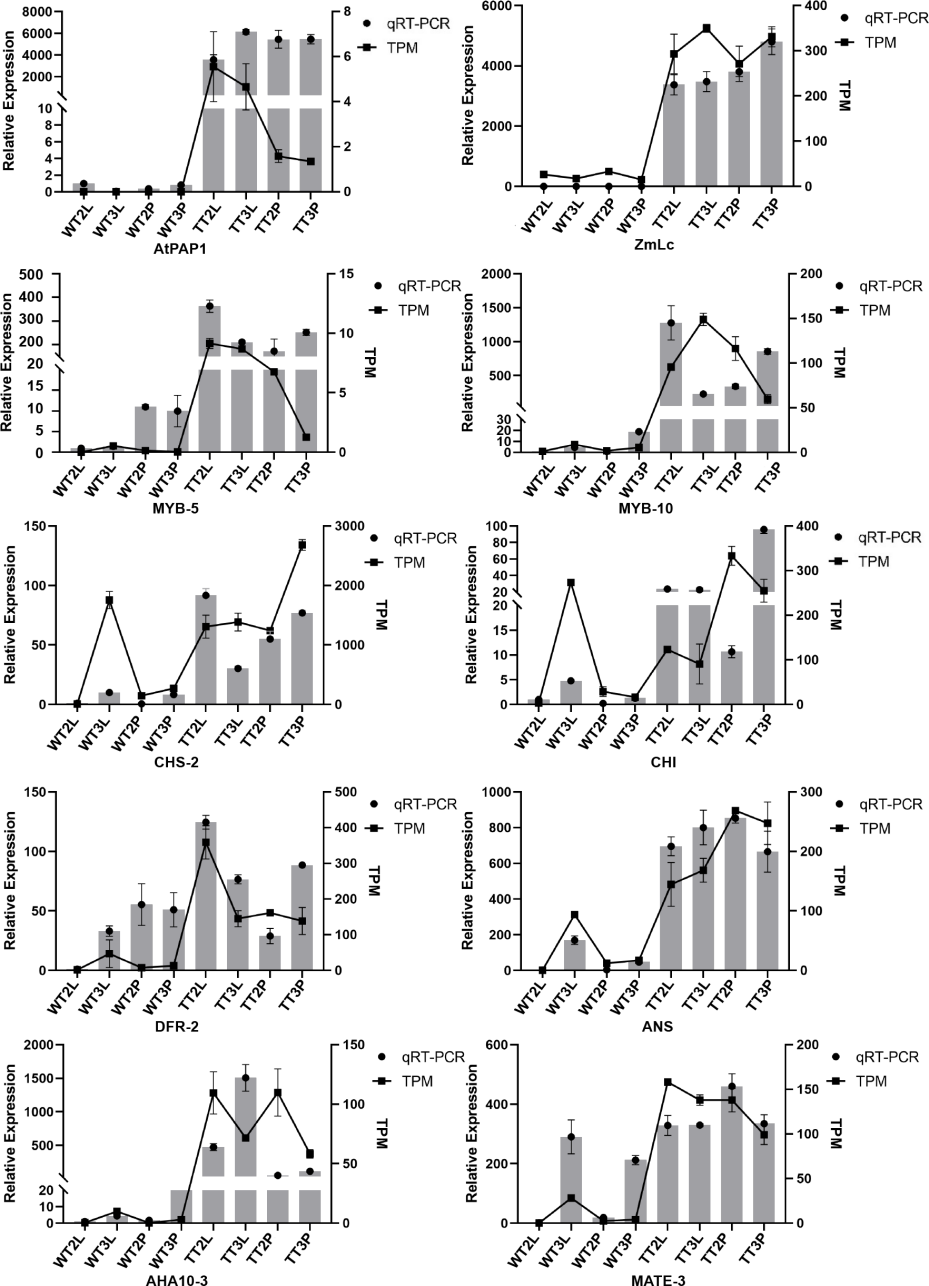
Validation of the expression of pigment-related genes in *C. bicolor* by qRT-PCR.

*AtPAP1*, *ZmLc* and eight key DEGs involved in anthocyanin biosynthesis were further analyzed using qRT-PCR to validate the transcript expression patterns of DEGs involved in anthocyanin biosynthesis. As a result, the high expression of *AtPAP1* and *ZmLc* in transgenic plants and the low expression detected in wild-type plants verified the reliability of the transgenic strains from the transcriptional level. Moreover, the expression pattern of the DEGs was consistent with the transcriptome data, and these in the wild-type T3 petioles were also significantly expressed (**Figure 6**). Furthermore, the high transcription level of *ANS* and *MATE-3* may directly lead to the color formation in *C. bicolor* leaf.

## 4 Discussion

### 4.1 Cyanidin-3-*O*-glucoside, cyanidin-3- *O* -rutinoside and peonidin-3-*O*-rutinoside are the main anthocyanins in the leaves and petioles of *C. bicolor*

Anthocyanins are key metabolites for coloration in plant organs, contributing to the purple leaves and the purple skin (Zhang et al., 2014a; Horiuchi et al., 2020; Tang et al., 2020). There are numerous species of anthocyanins present in colorful plants, with six of them being the most well-known, namely pelargonidin, cyanidin, delphinidin, peonidin, petunidin and malvidin. The molecular structures and chemical characteristics of these anthocyanins have been extensively investigated. In particular, cyanidin is the major contributor to the formation of red-purple pigments; delphinidin to blue-red or purple pigments; and pelargonidin to orange and red pigments (Khoo et al., 2017).

The colors of *C. bicolor* are diverse and the formation of color patches is closely related to the variation of anthocyanins and content. The anthocyanins in the *C. bicolor* have not been sufficiently identified or quantified. In our study, anthocyanins mainly accumulates in the vegetative organs of *C. bicolor* leaves and petioles, we detected and quantified the composition and content of anthocyanins in *C. bicolor*. The results showed that six aglycones of anthocyanin were detected in the *C. bicolor* leaves and petioles, and the main anthocyanins in *C. bicolor* were cyanidin, which was dominated by cyanidin-3- *O* -glucoside and cyanidin-3- *O* -rutinoside (**Figure S1A**; **Table S2**). The next major anthocyanins present were predominantly peonidin-3-*O*-rutinoside (**Figure S1B**), with significant amounts of pelargonidin-3-*O*-glucoside and pelargonidin-3-*O*-rutinoside (**Figure S1C**). Additionally, delphinidin, petunidin and malvidin were also detected, albeit in low levels (**Figure S1D**). These findings corroborate the results of (Lei et al., 2017), who demonstrated that cyanidin, pelargonidin were the main components of the red spathe in Zantedeschia hybrid (family Araceae). Notably, this study revealed significant levels of peonidin (peonidin-3-*O*-rutinoside), which has a bluish color compared to cyanidin and pelargonidin. Thus, the combination of these anthocyanin glycosides resulted in a reddish-purple color for *C. bicolor*. Furthermore, the species and content of peonidin were barely affected by the interaction of *AtPAP1* and *ZmLc*.

### 4.2 Pelargonidin-3-*O*-glucoside and pelargonidin-3-*O*-rutinoside content contributes to color variation in transgenic lines

In this studies, 17 anthocyanins among WT2L vs. TT2L, WT2P vs. TT2P, WT3L vs. TT3L and WT3P vs. TT3P were identified according to the anthocyanin content in each sample, which indicated that these 17 anthocyanins contribute to the different colors in transgenic lines of *C. bicolor*. The content of pelargonidin-3-*O*-glucoside and pelargonidin-3-*O*-rutinoside varied greatly among the 24 samples, and next were cyanidin-3-*O*-sambubioside and cyanidin-3-*O*-sophoroside (**Figure 1G**; **Table S3**).

The transgenic *S. baicalensis* hairy root cultures exhibited a distinct color change to a red brick hue upon introduction of *AtPAP1* and *ZmLc* into *S. baicalensis*, respectively. However, this study focused on flavone metabolic components and could not ascertain the effect of these introductions on the anthocyanins pathway (Ha Park et al., 2021). *AtPAP1* was transferred into *Nicotiana tabacum* and several transgenic plants developed red/purple pigmentation in different tissues. Qualitative analysis of anthocyanin composition in transgenic material was cyanidin-3- *O*-rutinoside (He et al., 2017). Ectopic expression of *AtPAP1* in *Solanum nigrum* increases anthocyanin accumulation in both nutritional and reproductive tissues of transgenic plants. The induced anthocyanins components were concentrated in delphinidin, petunidin and malvidin (Chhon et al., 2020). These scientific results are different from the results of the present study, the regulation of anthocyanin metabolic synthesis and transport in different plants is complex and diverse.

Wild type materials display an interesting phenomenon in which the petioles of the T2/T3 period possess the same anthocyanins content as the leaves, and even higher, yet there is no visible color change. This is due to a combination of factors will influence the color formation of plants, including the type and content of anthocyanins, intermolecular auxiliary coloring, molecular accumulation effect, chelation of metal ions, and the pH value of the vacuole (Jiang et al., 2020). Not only that, the leaves and petioles of *C. bicolor* differ from the petals and belong to vegetative organs, and in terms of pigment composition, photosynthetic pigments (the chlorophyll and carotenoid) are expected to be taken into account. Unsurprisingly, there are many unsolved mysteries about the complex coloration mechanism of *C. bicolor* waiting for us to explore.

### 4.3 Co-expression of AtPAP1 and ZmLc transcription factors affects anthocyanin accumulation in *C. bicolor* by regulating anthocyanin synthesis and transport

Anthocyanin metabolic engineering has been applied to improve the color of ornamental plants, as traditional breeding techniques such as hybridization and mutation are not efficient for the purpose (Boutigny et al., 2020). To achieve this, the overexpression of heterologous transcription factor genes is essential for regulating anthocyanin synthesis. The transcription factors AtPAP1 and ZmLc are widely used in anthocyanin metabolic engineering. Besides expression studies of the individual genes, *AtPAP1* and *ZmLc* have been simultaneously expressed to increase anthocyandin production in *Arabidopsis* and *S. involucrata* (Sharma and Dixon, 2005; Qiu et al., 2013). There was no significant color change in *S. involucrata* when *AtPAP1* and *ZmLc* were transferred alone. However, anthocyanin accumulation significantly increased in the callus and tender branches of transgenic tissue when *AtPAP1* and *ZmLc* were co-expressed. Li et al. also transferred *ZmLc* into *C. bicolor*, and the color of the whole leaf was not changed. Herein, *AtPAP1* and *ZmLc* co-expression in *C. bicolor* yielded similar results as when *ZmLc* was transferred alone.

The overexpression of MYB and bHLH TFs enhances the expression of anthocyanin biosynthetic genes and anthocyanin accumulation in many plants. In this research, anthocyanin accumulation increased in the transgenic lines. The co-overexpression of *AtPAP1* and *ZmLc* can activate the key genes related to anthocyanin synthesis and transport, thus improving anthocyanin biosynthesis.

Herein, we screened 9 structural genes and 5 transport key genes through PPI analysis, involved in each step of anthocyanin biosynthesis and transport. These genes were highly expressed at the transgenic lines and WT-T3 stage. The *F3’5’H* gene was not found in the transcriptome data. These results suggest that the cyanidin and pelargonidin biosynthesis pathways are involved in anthocyanin biosynthesis and promote color formation in *C. bicolor*. This is consistent with the results of metabolomics.

AtPAP1 and ZmLc can also stimulate other transcription factors, including 5 MYB-TFs, one bHLH-4, one WRKY and one HD-ZIP. MYB-TFs can directly regulate anthocyanin biosynthesis or interact with bHLH and WD40 transcription factors for anthocyanin biosynthesis regulation. Among the 5 MYB-TFs screened, MYB-5 and MYB-10 were more important. Phylogenetic analysis showed that the derived amino acid sequence of MYB-5 was highly similar to subgroup 5 R2R3-MYBs. The first R2R3-MYB transcription factor discovered to regulate anthocyanin biosynthesis is C1 (Colorless-1) in maize, belonged to subgroup 5 R2R3-MYBs, which specifically regulates the expression of *CHS* and *DFR* through light dependent regulation (Cone et al., 1993). And thus MYB-5 may act in the same manner in secondary metabolism. ZmC1 belongs to R2R3-MYB TFs, which can be classified into subgroups based on phylogenetic relationships and short signature motifs that are conserved within and unique to each subgroup (Tang et al., 2020). ZmC1 in subgroup 5 R2R3-MYBs, while TrMYB133 and TrMYB134, which MYB-10 homologous to subgroup 4 R2R3-MYBs (**Figure 5D**). These subgroup 4 R2R3-MYBs can be further classified into the ‘FaMYB1-type’ and the ‘AtMYB4-type’ (LaFountain and Yuan, 2021). The ‘FaMYB1-type’ regulates the flavonoid pathway, including crucial steps of anthocyanin biosynthesis, while the ‘AtMYB4-type’ regulates the general phenylpropanoid pathway leading to the production of phenolic acids and lignans (Ma and Constabel, 2019). TrMYB133 and TrMYB134 belonged to ‘FaMYB1-type’ can inhibit the activity of anthocyanin-regulated and proanthocyanidin-regulated MBW complexes, respectively (Albert, 2015). It is speculated that MYB transcription factors may be responsible for the significant increase in proanthocyanidin content of transgenic materials observed in this study, as they are known to regulate both anthocyanins and proanthocyanidins.

## 5 Conclusion

Constitutive promoter and heterologous expression of transcription factors have been employed to effectively improve the color presentation in *C. bicolor* petioles and leaves, with the aim of saving certain means of regulating biosynthesis and metabolism in this species. Although significant up-regulation of upstream regulatory transcription factors, as well as downstream key structural genes and transporter genes in the transgenic material was observed, no significant color changes were observed in the transgenic leaf margins. This indicates that the regulation of anthocyanin synthesis in *C. bicolor* is different in leaves and petioles, and that the mechanism of leaf color formation is complex, making commonly used genes insufficient for the targeted regulation of leaf spot in foliage plants. Therefore, further optimization of metabolic engineering strategies is needed to achieve targeted color improvement in ornamental plants. To this end, more efficient genes involved in anthocyanin biosynthesis and transport should be identified. For example, recent studies have demonstrated that MYB-TFs can regulate anthocyanin pigmentation in leaves; Wang et al. showed that the classic *Medicago truncatula* leaf anthocyanin spot trait depends on two R2R3-MYB paralogous regulators (RED HEART1 (RH1) and RH2) using *M. truncatula* leaf marking as a model (Wang et al., 2021). Further studies should be conducted to gain a better understanding of the mechanisms underlying the regulation of leaf spot in *C. bicolor*, and to identify more targeted and effective genes. In this study, MYB-5 and MYB-10 were found to have important roles in the regulation of anthocyanin synthesis in *C. bicolor*, and further in-depth functional studies can be conducted.

## Data availability statement

The data presented in the study are deposited in the National Center for Biotechnology Information (NCBI) repository, accession number PRJNA976473; https://www.ncbi.nlm.nih.gov/bioproject/PRJNA976473.

## Author contributions

SS and XY designed the research. AL, YH, and XL analyzed the data. JX and GG analyzed gene expression. XY, AL, and YH wrote the article. All authors contributed to the article and approved the submitted version.

## Supplementary material

Supplementary Figure 1 Anthocyanin composition statistics of *C. bicolor*.

Supplementary Figure 2 Protein sequence alignment of MYB-5 and MYB-10.
