# Supplementary figures and images for: Enhancement of the anthocyanin contents of Caladium leaves and petioles via metabolic engineering with co-overexpression of AtPAP1 and ZmLc transcription factors
Source: Front Plant Sci. 2023 Jun 21;14:1186816. doi: 10.3389/fpls.2023.1186816 (PMC10320811; doi:10.3389/fpls.2023.1186816)

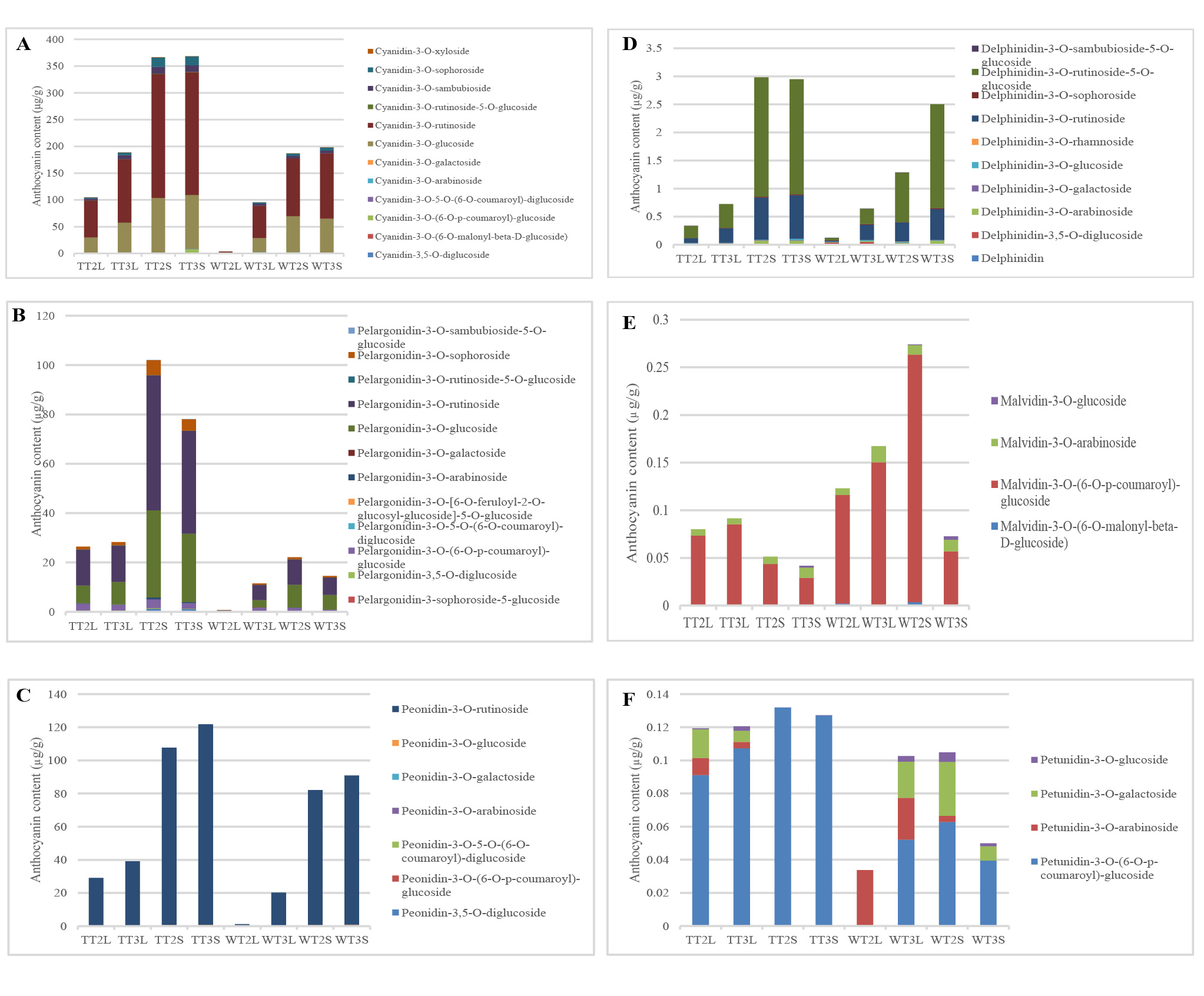

Supplement: Supplementary Figure 1 — Anthocyanin composition statistics of C. bicolor. [file Image_1.jpeg]

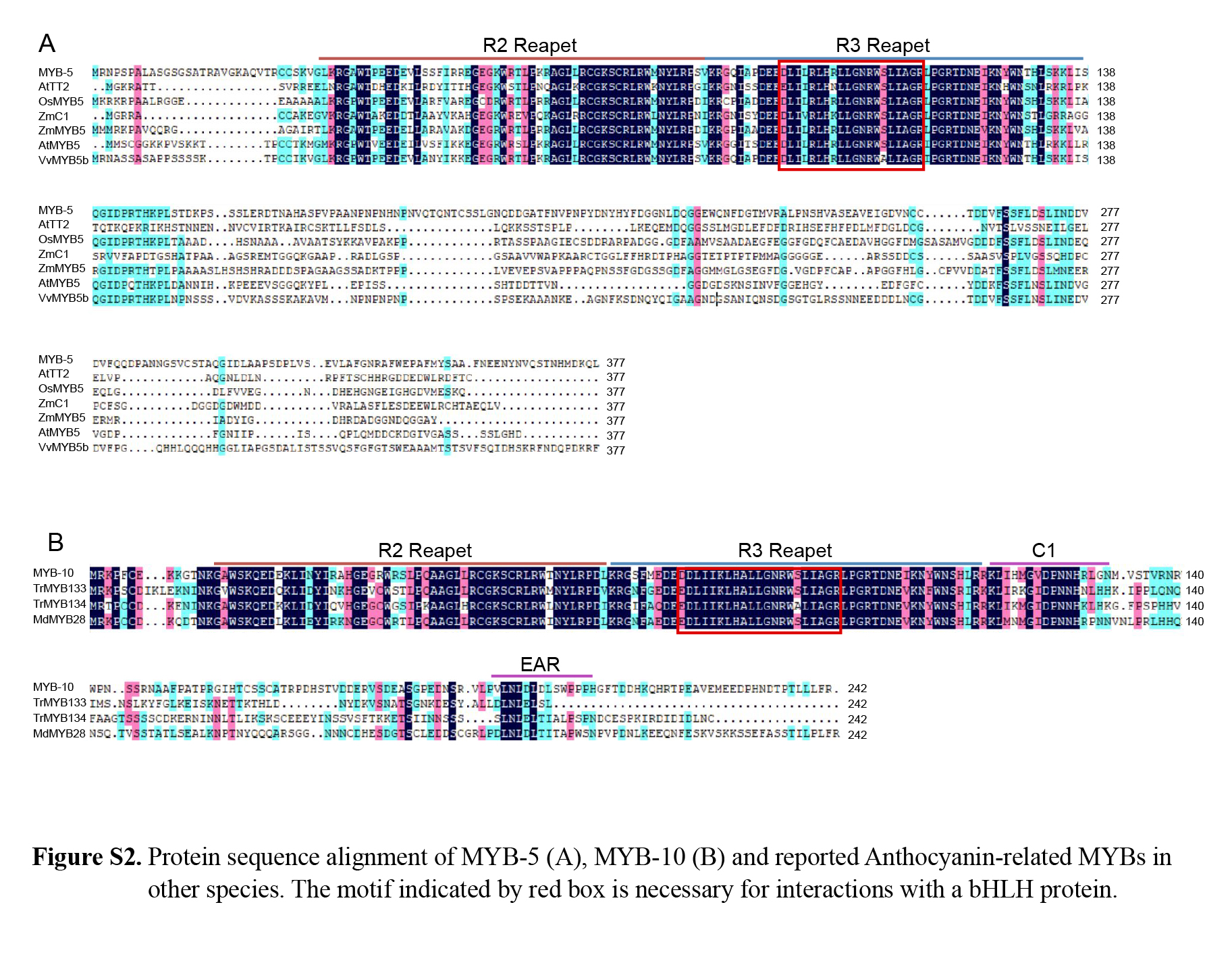

Supplement: Supplementary Figure 2 — Protein sequence alignment of MYB-5 and MYB-10. [file Image_2.jpeg]
