# Supplementary material for: Enhancement of the anthocyanin contents of Caladium leaves and petioles via metabolic engineering with co-overexpression of AtPAP1 and ZmLc transcription factors
Source: Front Plant Sci. 2023 Jun 21;14:1186816. doi: 10.3389/fpls.2023.1186816 (PMC10320811; doi:10.3389/fpls.2023.1186816)
Supplement: Supplementary file 3 [file Table_1.docx]

**Method S1. The plant binary expression vector (pC1300-*PAP1*+*Lc*)**


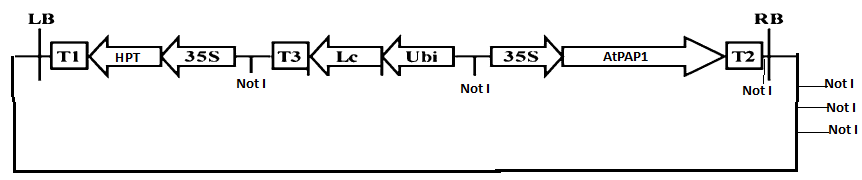


**AtPAP1Not I**

Ctggagggttcgtccaaagggctgcgaaaaggtgcttggactactgaagaagatagtctcttgagacagtgcattaataagtatggagaaggcaaatggcaccaagttcctgtaagagctgggctaaaccggtgcaggaaaagttgtagactaagatggttgaactatttgaagccaaatatcaagagaggaaaacttagctctgatgaagtcgatcttcttcttcgccttcataggcttctagggaataggtggtctttaattgctggaagattgcctggtcggaccgcaaatgacgtcaagaattactggaacactcatctgagtaagaaacatgaaccgtgttgtaagataaagatgaaaaagagagacattacgcccattcctacaacaccggcactaaaaaccaatgtttataagcctcgacctcgatccttctcagttaacaacgactgcaaccatctcaatgccccaccaaaagttgatgttaatcctccacgccttggacttaacaccaataatgtttgtgacaataatatcatatacaacaaagataagaagaaagaccaactagtgaataatttgattgatggagataatatgtggttagagaaattcctagaggaaagccaagaggtagatatgttggttcctgaaacgacgacaacagaaaaaggggacaccttggcttttgacgttgatcaactttggagtcttttcgatggagagactgtgaaatttgattag

**Lc (GenBank: M26227.1)**

atggcgctttcagcttcccgagttcagcaggcggaagaactgctgcaacgacctgctgagaggcagctgatgaggagccagcttgctgcagccgccaggagcatcaactggagctacgccctcttctggtccatttcagacactcaaccaggggtgctgacgtggacggacgggttctacaacggcgaggtgaagacgcggaagatctccaactccgtggagctgacatccgaccagctcgtcatgcagaggagcgaccagctccgggagctctacgaggccctcctgtcgggcgagggcgaccgccgcgctgcgcctgcgcggccggccggctctctgtcgccggaggacctcggcgacaccgagtggtactacgtggtctccatgacctacgccttccggccaggccaagggttgcccggcaggagtttcgcgagcgacgagcatgtctggctgtgcaacgcgcacctcgccggcagcaaagccttcccccgcgcgctcctggccaagagcgcgtccattcagtcaatcctctgcatcccggttatgggcggcgtgcttgagcttggtacaactgacacggtgccggaggccccggacttggtcagccgagcaaccgcggctttctgggagccgcagtgcccgagctccagcccgtcaggacgagcaaacgagaccggcgaggccgcagcagacgacggcacgtttgcgttcgaggaactcgaccacaataatggcatggacgacatagaggcgatgaccgccgccgggggacacgggcaggaggaggagctaagactaagagaagccgaggccctgtcagacgacgcaagcctggagcacatcaccaaggagatcgaggagttctacagcctctgcgacgaaatggacctgcaggcgctaccactaccgctagaggacggctggaccgtggacgcgtccaatttcgaggtcccctgctcttccccgcagccagcgccgcctccggtggacagggctaccgctaacgtcgccgccgacgcctcaagggcacccgtctacggctctcgcgcgacgagtttcatggcttggacgaggtcctcgcagcagtcgtcgtgctccgacgacgcggcgcccgcagcagtagtgccggccatcgaggagccgcagagattgctgaagaaagtggtggccggcggcggtgcttgggagagctgtggcggcgcgacgggagcagcacaggaaatgagtggcactggcaccaagaaccacgtcatgtcggagcgaaagcgacgagagaagctcaacgagatgttcctcgtcctcaagtcactgcttccgtccattcacagggtgaacaaagcgtcgatcctcgccgaaacgatagcctacctcaaggagcttcagagaagggtgcaagagctggagtccagtagggaacctgcgtcgcgcccatccgaaacgacgacaaggctaataacaaggccctcccgtggcaataatgagagtgtgaggaaggaggtctgcgcgggctccaagaggaagagcccagagctcggcagagacgacgtggagcgccccccggtcctcaccatggacgccggcaccagcaacgtcaccgtcaccgtctcggacaaggacgtgctcctggaggtgcagtgccggtgggaggagctcctgatgacgcgagtgttcgacgccatcaagagcctccatttggacgtcctctcggttcaggcttcagcgccagatggcttcatggggcttaagatacgagctcagtttgctggctccggtgccgtcgtgccctggatgatcagcgaggctcttcgcaaagctatagggaagcggtga
